# Medical Superstitions

**Published:** 1899-09

**Authors:** D. W. B. Conway

**Affiliations:** Ex-Physician and Surgeon to Virginia Polytechnic Institute; President Board of Health City of Athens, Ga.


### MEDICAL SUPERSTITIONS.

BY D. W. B. CONWAY, M.D.,

Ex-Physician and Surgeon to Virginia Polytechnic Institute; President Board of Health City of Athens, Ga.

The wheels of time are in constant motion, and in their onward march civilization, like the throbbing of the human heart, is keeping time. Notwithstanding we are living in an enlightened age, when we examine into the science of medicine many of our people are yet in a state of barbarism, and many superstitious ideas find credence among some of our more enlightened people. This is a world of change and progress. All the arteries of commerce, like the subterranean streams, are rapidly moving forward, dividing and subdividing into numerous ramifications, until they too are lost in the great ocean of unrest.

But while we recognize the fact that everything is onward in its course, the brightest recollections, the sweetest thoughts are often those that flash with a glance backward; and we love to linger over the memories of by-gone days, even in the rubbish of time, affording often amusement in matters medical.

To the masses medicine is a mystery, and not appreciated as it should be. Whether due to ignorance or superstition, it is a weakness of many to love the sensational, and they are often willing to be gulled by the charlatan—the long-haired healer from the Woolly West.

Our neighbors know more about our business than we do; they sometimes say to our patients: "Why, the doctor don't know anything about curing rheumatism; carry a buckeye or a piece of sulphur in your pocket for a while, and you'll soon be well."

Who does not remember the advice given us, when we were young, by our old colored mammy: "Don't let the moon shine into your face child while you are asleep, for it will sure make you crazy." The word lunacy may be significant of the above, and possibly originated from such suggestions. Our offspring are often weaned by the phases of the moon. With many parturition, as well as the catamenial flow, is often determined by the moon's phases. The soothing effect of the moonlight, I imagine, rather tends to soften and subdue the harsher sentiments of our human nature, and quiet, rather than excite the brain into mental hallucination. "Until the silver notes do touch and kiss the moonlight waves and charm the lovers wandering mid the vine-clad hills."

Heredity seems to be a potent factor in the preservation and dissemination of medical superstition, and we, as physicians, daily gather from those to whom we minister many quaint, amusing, and ridiculous notions from the superstitious.

The following are examples:

*For Thrash:* Get up soon in the morning; do not speak a word to any one until you have found nine sow-bugs; put them into a poke; tie around the child's neck.

*For the same:* Get some one whose name has not been changed by marriage, to blow her breath into the child's mouth.

*For Liver Grown:* Pass the child rapidly through a horse-collar, and then underneath the round of a chair; grease it with a marrow of a jawbone, and put to bed.

*For the same:* Take the child up gently, turn your back towards the north, and then swing the child slowly towards the west, south, and east three times in the name of the Father, Son, and Holy Ghost.

A doctor has many opportunities in his daily rounds among his patients of gathering the views and notions of the people about medicine and surgery. One asked me a few days ago, when I told her the uterus had been removed: "Did you put it back again?"

The charlatan controls a part of every community, and is found accomplishing his ends by repeating some inspired words over the patient, these words having been received by him in some mysterious way; but the words failed to accomplish a cure unless accompanied by blowing the breath upon the part affected. This reminds me that many think that the whole of medicine consists in wind, and such a doctor is satisfied that he knows enough when he knows how to blow.

Human superstition, as far back as the days of Hippocrates, has been a fruitful source of human error. We are told by Soranus, who wrote the life of Hippocrates, that honey proved an efficient remedy in aphtha of children, and he explains the matter very gravely by saying that the honey was obtained from a hive of bees near the tomb of Hippocrates.

*For Shingles:* Rub the part affected with the blood from the tail of a black cat.

*For Burns:* Get a man who is the seventh son to blow his breath upon the burn.

*For Wens:* Look at the new moon; pick up a bone, with which rub the wen and repeat the following: "What I look at increase, and what I rub decrease."

*For Toothache:* Get a splinter from a tree that has been struck by lightning; pick the tooth until you get blood upon the splinter, then bury it under the drip of a house.

*For Headache:* Bore a hole in a tree, put into it a lock of your hair and plug up the hole.

*Rupture*: Eat one dozen eggs that were laid on Thursday before Easter.

The negro race is well known among us for their superstitious ideas. Some of them believe that by an unknown cause they can be conjured; and unless removed by the party conjuring, they will pine away and die.

This race is scarcely more superstitious than our own, and it abundantly illustrates what I wish to show, that superstition can only exist to any great extent among the most ignorant, while credulity diffuses itself through the minds of all classes.

"In the opinion of the ignorant multitude," says Lord Bacon, "witches and impostors have always held a competition with physicians."

We all know that new remedies are filling our *materia medica*, and being rapidly replaced by others. This is done as much from fashion as from the emergencies that arise from the change of diseases.

Fashion has its votaries in medicine as well as in dress. It is said that Queen Anne was subject to hypochondriacal attacks, which her physicians called "spleen vapors," and advised certain confections and pearl cordial to be at once prescribed. Soon after a very noted work was published, treating upon nervous diseases and dyspepsia, and the whole fashionable world became dyspeptic.

*To Remove Warts*: Rub the wart with a snail until the snail has been crushed; take the remaining crushed portion, stick a pin through it, and hang it in such a place that the sun can shine upon it when it sets.

*Same:* Prick the wart with a pin; stick the pin through a bean leaf, then bury the leaf.

*Same:* Between two flat rocks place three buds of mullein, and rub until well bruised; take the bruised mullein and rub the wart, and then put in a dry place.

*For Leprosy:* Slay an innocent child or virgin, collect the blood, and apply freely over the patient.

*For Epilepsy:* Collect the blood from an executed criminal and apply to the patient.

In the early stages of society women were the doctors. While the men fought and hunted, the women gathered the herbs and decocted salves for their wounds. The older women were selected as having a superior skill for the profession. The "Wise Woman," with her kettle, cooking her mysterious broth, adding ingredient after ingredient—the more the rarer—inspired not only hope but fear. Roman matrons were often accused, and even convicted, of poisoning by their decoctions. It was carried on to such an extent, until the name of "poison maker" was given the preparer of the magic medicine.

An African priest and the medical man is one and the same, says Dr. Livingstone, and makes the clouds to give out rain by the following:

℞ Charcoal made of burned bats,
A lion's heart,
A calculus from a cow's stomach,
A serpent's skin and vertebræ,
Herbs, q. s.

Made into an infusion, the vapor from which rises and causes rain.

*Hemorrhage:* Repeat the sixth verse in the sixteenth chapter of Ezekiel, thus: "And when I passed by thee and saw thee polluted in thine own blood, I said unto thee when thou wast in thy blood, Live; yea, I said unto thee when thou wast in thy blood, Live."

*Same:* Place a basin of water under the bed, and also an axe with the sharp edge turned up towards the patient.

*Same:* Put a bunch of keys down the back, then go into a chimney corner, take out your knife, and pronounce the patient's name aloud as you open the knife, then repeat the name of the Father, Son and Holy Ghost.

*Bonefelon:* Catch a mole, and hold it up over your head and squeeze it in your hand until it dies.

*Hydrophobia:* Write the following on a slip of paper: "Box, mox, amox, armox," roll it up into a ball and swallow it.

*Stone in the Bladder:* Take three sow-bugs, dry and powder them, and give what will lie on the point of a knife, three times a day.

SUPERSTITIONS IN CHINA.

"Report of the Health of Wenchow," compiled by Dr. D. J. McGowen:

Epidemic frenzies are very common in China, the most conspicuous recent instance being the terror created in 1876 by the supposed supernatural clipping of queues. Sorcerers are in the habit of scattering charmed bits of paper, representing men, which are the means of disseminating evil spirits throughout the community. When one of these spirits enters a house he proceeds to cut off the queues of the inmates, and the sorcerer, on retaining possession of this, can evoke at will the soul of its owner, which he is able thereafter to use as a servile demon, while the man dies from the loss of his spirit. The only cure for the sufferer is to cut off an inch or two more of his hair, and keep it for eighty days soaking in a cesspool, thus severing the mysterious connection between his head and the portion of hair in possession of the sorcerer. But for prevention, reliance is placed on amulets and charms.

In 1876, the Governor of Kiangsee issued a proclamation, with a charm of his own invention, to be placed over the doors of dwellings, or to be worn as amulets. He further recommended an anathema, attributed to Tas Tsze, the founder of Tavism, which was to be chanted while copying it on yellow paper with the blood of a cock mixed with vermilion, the paper being then burned and the ashes swallowed. There was scarcely a house door that was not protected by a charm, and scarcely an individual who did not wear an amulet on cap or sleeve.

*Atrophy:* Go to the person performing the cure nine mornings before sunrise; rub the part affected with a rock; lay the rock down after passing it over the left shoulder in the name of the Father, Son and Holy Ghost.

*Pain, Colic, etc.:* Repeat the name of the person, and say the following: "Lucky is the day; lucky is the hour; lucky is the minute that it happened; pain, leave thyself and fly into the air, by the help of the Almighty. Amen."

*Goiter:* Rub the goiter over three times with the hand of a dead person.

*Neuralgia:* Get a child to rub its hands seven times over a barrel, and then apply them gently to the part affected.

*Atrophy:* Go before sunrise to a cedar bush, break off a twig, say to it, "no thanks to you." Walk backwards in a different direction from which you came until you stumble over wood or stone, and then bury the twig.

*To Stop Blood:* Take a bullet that has been shot into a beef, punch a hole in it, pass a string through it, and tie it around the patient's neck.

*Croup:* Put a mole's foot or amber beads on a string, and tie it around the child's neck.

INDIAN CURE.

*Toothache:* Repeat the following: "Toothache, I bid thee come out of the marrow into the bone, out of the bone into the blood, out of the blood into the flesh, out of the flesh into the skin, out of the skin into the ground twenty-seven furlongs deep."

*To Remove the Placenta:* Hold a little salt in the left hand, double up the right hand into a funnel shape and blow through it.

*Remove a Tumor:* Repeat the following: "Flesh and bone; marrow and bone. The tumor is harder than any bone." Rub it three times with a spade, and throw the spade over the left shoulder-blade.

*Hemorrhage:* Let a child who has never seen his father rub his hands gently over the face of the patient.

*Rupture:* Split a sapling and drag the patient through its prongs, when the bowel will return to its proper place.

Cats have been objects of superstition from the earliest ages. In Egypt they were held in the highest reverence; temples were erected in their honor; sacrifices and devotions were offered up to them, and it was customary for a family, in which a cat died, to shave the eyebrows. In the middle ages they were regarded as the familiars of witches. The favorite shape of Satan was said to be that of a black cat, and the animal was an object of dread instead of veneration.

There was a belief among sailors that the frolics of a cat at sea portended a storm. Many people still prophesy rainy weather from a cat washing its face, and a cat on the house top was held to signify death. The foolish belief that a cat has nine lives has lead to the perpetration of great cruelty to this harmless and very useful domestic animal.

*For Congestion of Liver:* Get some of the water in which a corpse has been washed; give a teaspoonful three times a day.

*Measles:* The excrement from the sheep, made into an infusion, is said to be a sovereign remedy.

*Hooping-cough:* Let the father of the child go to a stream, and catch nine fish, spit into their mouths and throw them back into the water alive and return.

*Same:* A teaspoonful of mare's milk three times a day.

*To take out Fire:* Blow your breath on the place burned and say: "Fire loose yourself as Judas lost his color the night on which he betrayed our Savior."

*Same:* Blow on the place and repeat the following: "As angels are holy, so we shall be holy that fire shall have no power within our flesh."

*To Rearrange the Bones of a New-Born Baby's Head:* Put the thumb of the left hand into the roof of the child's mouth, with the right hand support the back of the child's head; jump it up and down three times, when the head will be rounded symmetrical.

*Dressing New-Born Baby:* Slip the dress on over the boy's feet, as it will make him bashful if put on over his head; and just the reverse when dressing a girl. Do not cut the fingernails until they are one year old or it will make them steal.

*Remove Warts:* Rub them with milk-weed; then give the patient a rusty nail, and turn your back when he throws it at you, and leave without looking back. Bury the weed, and when it rots the warts are gone.

We were often led to believe that these and other superstitious customs were unworthy of consideration by those of education and refinement; but when we look upon the other side of the question it is impossible for us to form any accurate conception of the hypothesis upon which these customs originated, and no doubt the cause upon which these signs and tokens were established ceased to exist, while the practice still remained.

